# Magnetic Halloysite Nanotube-Based SERS Biosensor Enhanced with Au@Ag Core–Shell Nanotags for Bisphenol A Determination

**DOI:** 10.3390/bios12060387

**Published:** 2022-06-02

**Authors:** Sen Li, Defu He, Shuning Li, Ruipeng Chen, Yuan Peng, Shuang Li, Dianpeng Han, Yu Wang, Kang Qin, Shuyue Ren, Ping Chen, Zhixian Gao

**Affiliations:** 1 Tianjin Key Laboratory of Risk Assessment and Control Technology for Environment and Food Safety, Tianjin Institute of Environmental and Operational Medicine, Tianjin 300050, China; lisen2021@foxmail.com (S.L.); hedefu0919@gmail.com (D.H.); lishuning121@163.com (S.L.); chenruipeng2016@163.com (R.C.); dalidao@139.com (Y.P.); liza3320@163.com (S.L.); 15210520025@126.com (D.H.); wangyuyu9210@163.com (Y.W.); qinkang2020@foxmail.com (K.Q.); renshuyue2018@163.com (S.R.); 2 School of Food Science and Engineering, Jilin Agricultural University, Changchun 130118, China; 3 State Key Laboratory of Food Nutrition and Safety, Tianjin University of Science & Technology, Tianjin 300457, China

**Keywords:** surface-enhanced Raman scattering (SERS), aptasensor, bisphenol A, core–satellite assemblies

## Abstract

Bisphenol A (BPA) has emerged as a contaminant of concern because long-term exposure may affect the human endocrine system. Herein, a novel aptamer sensor based on magnetic separation and surface-enhanced Raman scattering (SERS) is proposed for the extremely sensitive and specific detection of trace BPA. Moreover, the capture unit was prepared by immobilizing thiolated (SH)-BPA aptamer complementary DNA on AuNP-coated magnetic halloysite nanotubes (MNTs@AuNPs), and SH-BPA aptamer-modified Au@4-MBA@Ag core–shell SERS nanotags acted as signal units. By the complementary pairing of the BPA aptamer and the corresponding DNA, MNTs@AuNPs and Au@4-MBA@AgCS were linked together through hybridization-ligation, which acted as the SERS substrate. In the absence of BPA, the constructed aptamer sensor generated electromagnetic enhancement and plasmon coupling to improve the sensitivity of SERS substrates. Owing to the high affinity between BPA and the aptamer, the aptamer probe bound to BPA was separated from the capture unit by an externally-induced magnetic field. Thus, the Raman intensity of the MNTs@AuNP-Ag@AuCS core–satellite assemblies was negatively correlated with the BPA concentration. High sensitivity measurements of BPA might be performed by determining the decline in SERS signal strength together with concentration variations. The proposed aptasensor is a promising biosensing platform for BPA detection.

## 1. Introduction

Bisphenol A (BPA), also known as 2,2-bis(4-hydroxyphenyl) propane, has a chemical structure similar to that of endogenous estrogens and can mimic or interfere with endogenous estrogen action to exert estrogenic effects; it is a typical endocrine-disrupting chemical [1]. BPA is a commonly used industrial compound, mainly used in the manufacturing of polycarbonates and resins and other products required for daily life, such as medical equipment and sports items. Refreshments are protected from direct contact with metals using epoxy resins as an inner coating for cans, commonly used in food and beverage containers, including milk bottles, and as additives to other plastics [2]. Studies have shown that polymerized BPA molecules found in epoxy resins, bound with ester bonds, can leach into water and food containers and baby bottles under high-temperature, acidic, or alkaline conditions and can induce chronic conditions such as diabetes, congenital disabilities, kidney and cardiovascular diseases, obesity, endometrial cancer, breast cancer, and sexual dysfunction after exposure for a long time [3,4,5,6,7,8].

There are various analytical approaches to detect BPA, including gas chromatography–mass spectrometry, liquid chromatography–mass spectrometry, high-performance liquid chromatography (HPLC), and magnetic solid-phase extraction [9,10,11]. Although these procedures are sufficiently accurate, they have several drawbacks, including expensive instruments, laborious pretreatment, a requirement for trained operators, and high detection expenses. Other recently developed approaches such as enzyme-linked immunosorbent assay [12], fluorescence technique [13], colorimetry [14], microfluidic devices [15], and electrochemical methods [16] have also been used for the rapid detection of BPA. 

Spectroscopic methods have recently received considerable research interest because of their high sensitivity, easy operation, multiplexing capability, and suitability for the fabrication of portable devices. Among these spectroscopy methods, surface-enhanced Raman scattering (SERS) is a promising strategy because it provides a unique spectroscopic fingerprint with non-destructive and ultrasensitive characteristics [17]. The magnitude of electromagnetic (EM) and chemical enhancements (CM) affect SERS signal augmentation, with EM having a key influence [18,19]. The long-range EM effect depends on the distance to the core surface, whereas the short-range chemical effect depends on atomic-level roughness [20]. 

SERS is based on the substantial Raman signal amplification that occurs when localized surface plasmon resonances (LSPRs) are excited. The dominant enhancement mechanism of SERS has been attributed to the giant electromagnetic field enhancement (hot spot effect) upon the excitation of LSPR, which can additionally enhance the intensities and sensitivities of the signal [21]. Theoretically, depending on the plasmonic nanomaterials used, the total SERS enhancement factor may approach 10^14^ [22]. Although SERS performance may be realized by enhancing the electromagnetic resonance characteristics of nanoscale noble metal surfaces, the preparation of SERS substrates with reliable, uniform, and high enhancement factors is still a challenge.

Halloysite nanotubes (HNTs) are naturally occurring, two-layered (1:1) silica-aluminate material. Various surface charge characteristics on the exterior and interior of HNTs can be readily and effectively functionalized for the specific loading of multiple molecules. To boost SERS sensitivity, HNTs can be used as scaffolds to carry Au nanoparticles [23]. Therefore, the functionalization of HNTs plays a pivotal role in the construction of SERS substrates. The functionalized AuNP-coated magnetic HNT (MNTs@AuNPs) substrates have a potent LSPR effect, thus forming a defined plasmonic field “hot spot” with a fine-tunable interparticle gap after magnetic separation. Coupling SERS tags with MNTs@AuNPs created plasmonic dimers to extend the SERS enhancement field at the hot spots. 

In this study, a thiolated aptamer probe for BPA was attached to the surface of 4-mercaptobenzoic acid (4-MBA)-embedded gold/silver core–shell nanostructures to form the signal unit. CoFe_2_O_4_@HNTs-AuNPs nanocomposites (MNTs@AuNPs) were fabricated using vacuum cycling and in situ reduction, which were modified with BPA aptamer complementary DNA to form capture units. The aptamer conjugated with Au@4-MBA@AgCS nanoparticles (signal unit) was hybridized with cDNA-modified MNTs@AuNPs (capture unit) to form a biosensing platform for BPA detection. The strengths of the novel aptasensor are as follows. (1) Considering its excellent interface properties, we filled magnetic CoFe_2_O_4_ beads into its inner surface to fabricate MNTs with abundant aluminol groups. Moreover, MNTs have been proposed as supports for generating a large amplification factor for Raman signals by coating HNTs with Au nanoparticles. (2) The Raman reporter molecule 4-MBA anchored between the Au core and Ag shell (Au@AgCS) successfully avoided interference with external conditions, which could greatly enhance the Raman intensity and detection sensitivity. (3) We integrated the signal units and capture units into the competing system to form core–satellite nanostructures, thus creating rich electromagnetic hot spots with a well-tuned interparticle distance after magnetic separation. Magnetic HNTs serve as Raman signal enhancers and magnetic enrichment materials. (4) Based on the specific binding affinity between the aptamer and BPA, analyte-controllable SERS hot spots at the nanogap between the core and satellite nanostructures could be created, improving the sensitivity of BPA detection. Upon conformational alteration induced by BPA aptamer-target recognition, the corresponding Raman intensity of the biosensor was negatively correlated with the BPA concentration following magnetic separation. Finally, we constructed an aptamer sensor with a SERS mode to detect BPA in real samples, which exhibited great potential for on-site detection.

## 2. Materials and Methods

### 2.1. Reagents and Materials

The reagents and chemicals were graded and used without further distillation. Shanghai Sangon Biological Engineering Technology & Services Co. Ltd. (Shanghai, China) provided DNA oligonucleotides. Gold (III) chloride trihydrate (HAuCl_4_·3H_2_O) and 3(2-carboxyethyl) phosphine hydrochloride (TCEP) were purchased from Aladdin Reagent Co. Ltd. (Shanghai, China). Halloysite clays (HNTs), silver nitrate (AgNO_3_), and BPA (C_15_H_16_O_2_) were acquired from Sigma-Aldrich (Shanghai, China). Magnesium chloride (MgCl_2_·6H_2_O) was obtained from Tianjin Jinke Fine Chemical Research Institute. 4-MBA and 10 × PBS buffer were purchased from Yuanye Biology Co., Ltd. (Shanghai, China). Ferric nitrate and cobalt nitrate hexahydrate were purchased from Rhawn Reagent Co. Ltd. (Shanghai, China). Glacial acetic acid, 3-aminopropyltriethoxysilane (APTES), and Tween-20 were obtained from Solarbio Co. Ltd. (Beijing, China). Ultrapure water acquired from a Millipore Milli-Q water purification system was employed throughout the experiments. The specific aptamer sequence of BPA was selected according to a previous report [18]. The DNA sequences were as follows: Apt: 5′-SH-TTTTTTTTTTTTTTGGGTGGTCAGGTGGGATAGCGTTCCGCGTATGGCCCA-3′; complementary Apt strand cDNA: 5′-SH- TTTTTTTTTTTTTGCGGAACGCTAT-3′.

### 2.2. Instruments and Equipment

The morphologies were observed using a transmission electron microscope (Talos F200X, FEI, Newark, DE, USA) and a scanning electron microscope (Zeiss Merlin, Oberkochen, Germany). A Zetasizer Nano ZS instrument was used to perform the dynamic light scattering measurements (Marlvern 3000, Southborough, UK). UV-vis spectra were recorded using a UV-vis spectrophotometer TU-1901 (Persee Instrument Co., Ltd., Beijing, China) with working wavelengths of 300–700 nm. The SERS measurements were conducted using a Raman spectrometer with a 785-nm laser at room temperature (i-Raman Plus, B&W Tek, Waltham, MA, USA). Each sample was excited at 25 mW over a 5-s acquisition time. All of the Raman spectra were collected between 700 and 1800 cm^−1^. 

A Nicolet iS 5 Frontier transform infrared spectrophotometer (Thermo Fisher Scientific, Waltham, MA, USA) was used to acquire FT-IR spectra with wave numbers ranging between 4000 and 500 cm^−1^. Liquid chromatography was performed using a Waters Alliance-2695 HPLC system.

### 2.3. Preparation of Magnetic HNTs@AuNPs Composites

#### 2.3.1. Preparation of MNTs

The HNTs (5 g) were blended with 50 mL of piranha solution (prepared by blending a 7:3 volume ratio of concentrated H_2_SO_4_ and 30% H_2_O_2_ solution). The dispersion was stirred under reflux for 1 h at 90 °C. Active HNTs were removed from the piranha mixture using centrifugation, rinsed with distilled water until the supernatant became neutral, and dried in a vacuum overnight at ambient temperature.

A 100-mL solution of metallic nitric acid was prepared by stirring 48.48 g of Fe(NO_3_)_3_·9H_2_O and 17.46 g of Co(NO_3_)_2_·6H_2_O in a mixture containing ethanol and water at a volume ratio of 40:60. Next, 2 g HNTs were suspended in 20 mL of the metal solution, and the Fe^3+^ and Co^2+^ dispersions were filled into the purified HNTs using vacuum circulation (where the concentrations of Fe^3+^ and Co^2+^ were 1.2 mM and 0.6 mM, respectively); this process was repeated thrice. Solid HNTs of the filled dispersions were dried at 60 °C for 12 h, and the dried product was denoted as MNTs.

#### 2.3.2. Preparation of MNTs@AuNPs 

First, 2 mg of dopamine was stirred in 50 mL (pH = 8.5) Tris-HCl buffer solution; then, 5 mL of 10 mg mL^−1^ purified MNTs was introduced, heated to 30 °C, and stored for 6 h. All black products were obtained, rinsed with ethanol, and dried for 12 h at 60 °C. Next, 50.0 mg of dopamine-modified MNTs was dispersed into 45.0 mL of ultrapure water; subsequently, 5 mL of 10.0 mM HAuCl_4_ solution was added along with 50 mM ascorbic acid (0.5 mL) to an ultimate concentration of 0.5 mM. The product was stirred at 30 °C for 3 h. Thereafter, the resulting solution was centrifuged and dissolved in 20.0 mL ultrapure water after cleansing. The resulting product was the MNTs@AuNP.

### 2.4. Preparation of AuNPs@4-MBA@AgCS

The AuNPs were produced by the reduction of HAuCl_4_ with sodium citrate as described previously [24]. The HAuCl_4_ solution (29.4 mM, 0.02 mL) was combined with 1 mL of HEPES buffer (pH 7.4) and shaken vigorously for 30 s before being put in the dark for 30 min. Modifying the saturation of HEPES can change the form and size of AuNPs. The combined solution was then added to the 4-MBA solution (20 mM, 0.005 mL), which was linked to AuNPs via Au-SH covalent bonds.

The Ag shells of Au@AgNPs were produced through the reduction of silver ions (Ag^+^) in a solution of L-ascorbic acid. AgNO_3_ solution (0.2 mL, 0.2 mM) was added to a 2-mL centrifuge cylinder. The centrifuge cylinder was filled with 1 mL of the produced AuNP solution and 0.03 mL of 100 mM L-ascorbic acid solution. The mixture was shaken vigorously for 3 s and then placed in the dark for 30 min. Au@4-MBA@AgCS was obtained at the end of the reaction and was stored at 4 °C.

### 2.5. Preparation of the SERS Aptamer Sensor for BPA Detection

#### 2.5.1. Functionalization of Au@4-MBA@AgCS with Aptamer

First, 1 mL of Au@4-MBA@AgCS was centrifuged twice at 8000 rpm for 5 min to eliminate any extra substances and was redispersed in deionized water. Next, the Au@4-MBA@AgCS solution was reacted with 50 μL of the newly prepared mPEG-SH solution (20 μM) for 20 min and then shaken vigorously. Finally, 1 mL of 0.01 wt% Tween-20 was added to resuspend the solution. The centrifugation followed by resuspension with mPEG-SH and Tween-20 step was performed thrice. The Au@4-MBA@AgCS-aptamer was obtained by adding 8 μL of 100 μM activated aptamer DNA and 50 μL of 20 μM MgCl_2_ to the mixture solution, aging for 1 h at ambient temperatures, and then resuspending by centrifugation with PBS thrice to remove the free aptamer.

#### 2.5.2. Functionalization of MNTs@AuNPs with Complementary DNA Strand

The DNA strands complementary to the aptamer were altered on the surface of the MNTs@AuNPs by forming Au–SH covalent bonds. Briefly, 10 μL of 10 mM TCEP was mixed with 10 μM of thiolated complementary DNA (SH-cDNA) in 0.5 × PBS buffer. After vigorous shaking for 10 s, the mixture was incubated at 37 °C for 1 h to activate the thiol-modified oligonucleotides. After the reduction with TCEP, 45 μL of MNTs@AuNPs was added to the mixture solution to obtain a final volume of 100 μL. This mixture was directly mixed with 900 μL n-butanol and quickly vortexed for a few seconds. After centrifugation at 8000 rpm for 1 min to eliminate additional n-butanol, the residue was resuspended in 100 μL of deionized water for further use.

#### 2.5.3. Preparation of the SERS Aptasensors 

MNTs@AuNP-Au@AgCS core–satellite assemblies were produced by mixing capture (MNTs@AuNPs-cDNA) and signal units (Au@4-MBA@AgCS-Apt) at various volume ratios and hybridizing for 1 h at 37 °C. After magnetic separation and two washes with ultrapure water, the SERS aptamer sensors were produced and kept at 4 °C to maintain biological activity and stability.

Under the optimized conditions, the specimens of multiple BPA concentrations (0, 0.005, 0.05, 0.5, 5, 50, and 500 ng mL^−1^) were tested using the SERS aptasensor at 37 °C for 90 min to ensure complete recognition between aptamers and BPA. Thereafter, the MNTs@AuNP-AgCS core–satellite assemblies were rinsed with deionized water and magnetically separated to obtain the SERS spectra. The SERS measurements were conducted using a Raman spectrometer with a 785-nm laser at room temperature (i-Raman Plus). Finally, for SERS detection, 5 µL of the mixture was poured onto a silicon wafer and allowed to dry naturally in the air. Each sample was excited at a power of 25 mW for 5 s. All of the Raman spectra were measured between 700 and 1800 cm^−1^. 

All of the experiments were repeated five times to confirm the results. To reduce ambiguity, the mean value was chosen. The standard deviations of five separate readings are indicated by error bars and shown in the figures. According to IUPAC recommendations, the limit of detection (LOD) of BPA was calculated using the signal-to-noise ratio (S/N = 3): LOD = 3 S_b_ /m, where S_b_ represents the standard deviation of five blank SERS readings and m is the analytical sensitivity projected as the slope of the calibration curve.

### 2.6. Specificity and Applicability Evaluation

The specificity of the Raman aptasensor was investigated in the absence of any target in the presence of 1 ng mL^−1^ estradiol (E2), 4-tert-butylpyridine (4-tBP), 4,4′-dihydroxybiphenyl (DOD), and diethylstilbestrol (DES). 

The recovery rates of the SERS biosensors were studied after spiking various concentrations of BPA into the actual samples. First, the PC bottles were selected as real samples and cut into pieces. The mixtures were dispersed in 100 mL of a 60% methanol–water solution; then, they were briefly (up to 2 h) sonicated for better redispersion in water. Subsequently, the samples were placed in a water bath at 80 °C for 48 h. Finally, a membrane filter (0.22-μm pore-size) was used to filter the samples, and the filtrate was collected for further analysis. For the detection of actual samples, different concentrations of BPA were added to the samples, which were then detected using the developed SERS aptasensors.

## 3. Results and Discussion

### 3.1. Detection Principle

**Scheme 1:**
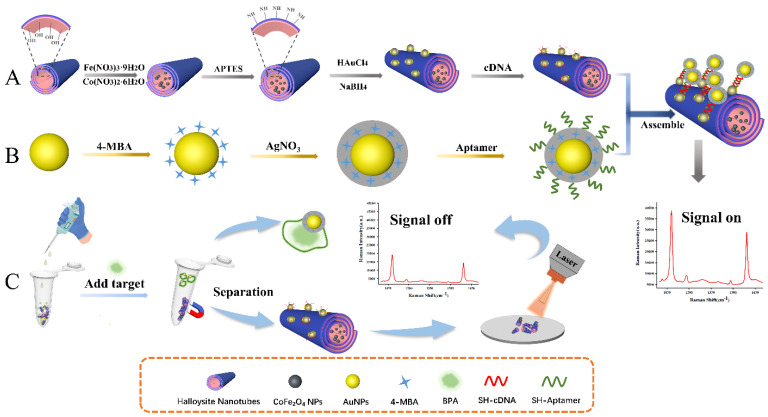
Schematic illustration of the magnetic halloysite nanotube-based SERS biosensor enhanced with Au@Ag core–shell nanotags for bisphenol A detection. (**A**) preparation of MNTs@AuNPs; (**B**) preparation of AuNPs@4-MBA@AgCS; (**C**) preparation of the SERS aptasensor for BPA detection.

A schematic depiction of the SERS biosensor production technique and detection principle is shown in Scheme 1. Here, we constructed a multifunctional magnetic HNT-based SERS substrate coupled with 4-MBA-embedded gold/silver core–shell nanostructures (Au@4-MBA@AgCS) as novel SERS tags. Owing to the unique structure of the outer surface of HNTs, AuNPs immobilized on MNTs (MNTs@AuNPs) were used as SERS substrates to detect 4-MBA, a typical model analyte embedded in gold/silver core–shell nanostructures for SERS performance evaluation. Thiol-modified cDNA (DNA that is partially complementary to the aptamer) was bound to the surface of the MNTs@AuNP composite by forming Au–S bonds. Next, a highly sensitive and selective SERS aptasensor was constructed to induce electromagnetic enhancements and a uniform enhancement of hot spots between plasmonic nanoparticles to increase the sensitivity of the SERS substrates. In the absence of BPA molecules, Au–Au dimerization generated the maximum electric field enhancement, which helped to enhance the LSPR effect at the hot spots. In the presence of BPA, aptamers on Au@4-MBA@AgCS attached to BPA molecules, allowing the signal unit to be released from the capture unit and the hot spots to fade away, resulting in a reduction in the 4-MBA SERS intensity following magnetic separation. With an increase in BPA concentration, the decrease in the Raman intensity revealed a negative linear correlation.

### 3.2. Structural Characterization of MNTs@AuNPs Composites

**Figure 1:**
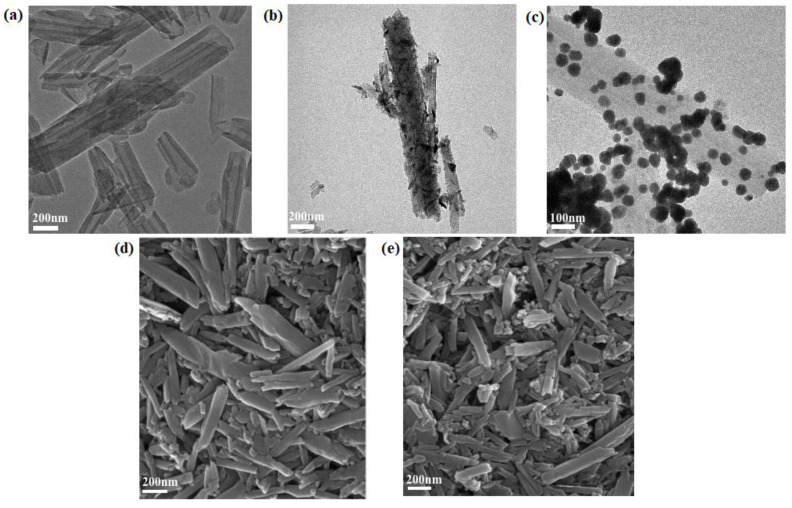
Transmission electron micrographs of HNTs (**a**), MNTs (**b**), and MNTs@AuNPs (**c**); scanning electron micrographs of HNTs (**d**) and MNTs (**e**).

Transmission electron microscopy (TEM) and scanning electron microscopy (SEM) were used to examine the structures and morphologies of the prepared materials. Figure 1a shows that the overall morphological and structural characteristics of the natural HNTs show a rod-like morphology with a tubular structure and submicron resolution. The inner diameter of the hollow lumen structure was 15–20 nm, whereas the outer diameter was approximately 30–50 nm. Scanning electron micrographs show the length of the HNT lumen to be 500–2000 nm, indicating the polydispersity of HNTs. Figure 1b indicates that CoFe_2_O_4_ nanoparticles can be firmly attached to the inner surfaces of the tubes and make the nanomaterials magnetic. TEM revealed well-dispersed AuNPs on the exterior surface of HNTs with no aggregation (Figure 1c). Notably, the natural HNTs (Figure 1d) have no obvious morphological changes when compared with CoFe_2_O_4_@HNT (Figure 1e). This indicates that the modification of the magnetic particles in the inner tube did not alter the structure of the pores of the HNTs. This also explains the decoration of the internal surfaces of HNTs with the magnetic CoFe_2_O_4_ nanoparticles. The above characterization indicates the formation of AuNP-modified CoFe_2_O_4_@HNTs. 

To investigate the magnetic properties of MNTs@AuNPs, hysteresis curves were plotted. The saturation magnetization intensity (MS) of MNT@AuNPs was 25.4 emu g^−1^ (Figure S1), which exhibited superparamagnetism, indicating that MNTs@AuNPs have good magnetic properties. In the actual magnetic separation test, it could be completely separated within 20 s under the action of the magnet, which could be used for the separation of the reporter probes to enhance the Raman signal further.

The FT-IR spectra of HNTs, MNTs, and MNTs-NH_2_ are displayed in Figure S2. The infrared absorption peaks at 3697 cm^−1^ and 3622 cm^−1^ are the vibrational peaks of the inner and outer hydroxyl groups, respectively, which are characteristic infrared peaks that discriminate HNTs from other mineral materials [25]. The infrared absorption peak at 3485 cm^−1^ is the stretching vibrational peak of the water O–H bond. After filling the interior of HNTs with CoFe_2_O_4_ magnetic nanoparticles (red line), a new infrared absorption peak appears at 580 cm^−1^, which is attributed to the generation of Fe–O and Co–O bonds. The internal hydroxyl vibrational absorption peak of HNTs disappeared because the growth of CoFe_2_O_4_ magnetic particles inside the HNTs destroyed the internal hydroxyl environment of the HNTs, indicating that CoFe_2_O_4_ magnetic nanoparticles were successfully grown on HNTs. The peaks detected at 2926 cm^−1^ and 2860 cm^−1^ of CoFe_2_O_4_@HNTs were attributed to C-H asymmetric and symmetric stretching vibrations, respectively, suggesting the presence of APTES grafted onto the CoFe_2_O_4_@HNTs. The peak position of the stretching vibration peak of the water O–H bond at 3485 cm^−1^ shifted toward a lower wavenumber, indicating the generation of N–H bonding groups (the peak positions of the symmetric and asymmetric stretching vibrations of the N–H bond are 3300 cm^−1^ and 3360 cm^−1^, respectively), which are attributed to modification by the aminosilylation reagent. The results showed that APTES, which is the only source of C–H and N–H, was effectively fused onto the HNTs.

Figure S3 shows the Zeta potentials of HNTs, HNTs@CoFe_2_O_4_, MNTs-NH_2_, and MNTs@AuNPs. It was found that the Zeta potential increased from −19.22 mV to −11.21 mV before and after embedding magnetic CoFe_2_O_4_ beads into the HNT’s inner cavity. Then, the surfaces of the MNTs were functionalized with -NH_2_ groups using APTES. MNTs possess a negatively charged surface with a Zeta potential of −11.21 mV, whereas MNTs-NH_2_ show a positively charged surface with a Zeta potential of +16.75 mV. The AuNPs were coated on the external layer of the HNTs via the in situ reduction of Au ions with the inclusion of tea polyphenols. The Zeta potential decreased from 16.75 mV to −8.44 mV, signifying that the gold nanoparticles were effectively altered on the surface of MNTs. The synthesized MNTs@AuNPs were used in the subsequent experiments.

The MNTs and MNTs@AuNPs powders were analyzed using X-ray powder diffraction to verify the existence of AuNPs adsorbed on the HNTs. Figure S4 shows the diffractograms before and after the loading of MNTs with AuNPs. The diffraction patterns of the MNTs@AuNPs were obtained, as indicated by the blue line. The peaks appearing in the original HNTs can be indexed to JCPDS card 29-1487, and the detected peak is consistent with the characteristic peak of 7 Å-dehydrated HNTs (Al_2_Si_2_O_5_(OH)_4_, aluminum silicate) [26,27]. Subsequently, when the AuNPs were fused on the external layer of AuNPs@HNTs (blue curve), a stronger diffraction peak at 2θ = 38.3° and three comparatively weaker peaks at 2θ = 44.4°, 64.6°, and 77.5° corresponded to the reference standards of Au (111), Au (200), Au (220), and Au (311) lattice planes, respectively [28,29,30]. These results established that AuNPs@HNTs were successfully prepared.

The chemical states of AuNPs immobilized on magnetic functionalized HNTs (MNTs@AuNPs) are observed in Figure S5; the strong double peaks of 84.6 ev and 88.3 ev correspond well to the Au 4f_7_ and Au 4f_5_ binding energy, respectively [31]. X-ray photoelectron spectra analysis demonstrated that the Au/APTES interface fosters strong metal–ligand chelation effects on the AuNPs to produce positively charged Au. In addition, the characteristic peaks of the C 1s and N 1s spectra (Figure S5) indicate that APTES was chemically bonded to the external layer of the MNTs to enable the deposition of Au nanoparticles on the MNTs. As shown in Figure S5e, the O 1s peak of the XPS spectrum displays a single peak that corresponds to the binding energy value of 532.1 eV, implying the bonding of O atoms with Al and Si in Al_2_Si_2_O_5_(OH)_4_. As shown in Figure S5f, the Si 2p high-resolution spectrum also exhibits a peak at 102.8 eV, which corresponds to the Si–O–Si bond. The single peak in the Al 2p spectrum (74.5 eV; Figure S5g) mainly originates from the Al–O bond. The high-resolution XPS scan curves for the O, N, C, Si, Au, and Al elements in MNTs and MNTs@AuNPs demonstrated that the Au nanoparticles could attach to the outer surface of MNTs to form the SERS active substrate.

### 3.3. Characterization of Au@4-MBA@AgCS Composites

**Figure 2:**
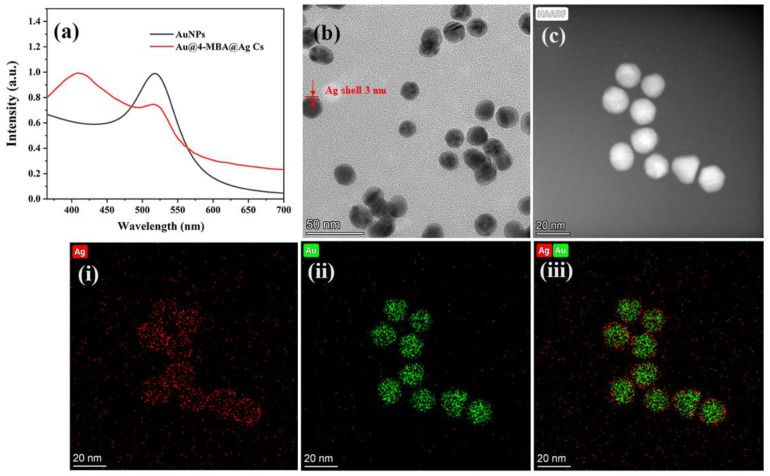
(**a**) UV-vis spectra of AuNPs and Au@4-MBA@Ag; (**b**) transmission electron micrographs of Au@4-MBA@Ag; (**c**) HAADF image of Au@4-MBA@Ag and the corresponding element mapping images (**i**–**iii**).

The formation processes of the Au@4-MBA@Ag core–shell nanostructure were investigated using UV-vis absorption spectroscopy, TEM, SEM, and mapping element analysis. The UV-vis spectra of AuNPs and Au@4-MBA@AgCS are shown in Figure 2a. AuNPs exhibited a typical absorbance band at 520 nm. The existence of a new band at 410 nm and a broader full width at half maximum indicated the formation of the Ag shell. This is because AuNPs and Au@4-MBA@AgCS have distinct SPR frequencies and the same particle size.

Figure 2b shows high-resolution transmission electron micrographs of Au@4-MBA@AgCS NPs. Au@4-MBA@AgCS had a 20-nanometer dimension and a 4-nanometer Ag shell thickness.

HAADF-STEM images and corresponding element mapping images were obtained to further confirm the formation of Au@4-MBA@AgCS. Given the enormous disparity in the atomic numbers of Ag and Au atoms, the HAADF-STEM images of the Au@4MBA@Ag nanoparticles reveal a brilliant core and gray shell layer structure, as shown in Figure 2c. However, the elemental mapping photos revealed the distribution of Ag on Au. Furthermore, Ag atoms (red) were found around the core of the Au atoms (green) on the surface, which is compatible with the Au core and Ag shell nanostructure. These observations indicated that the highly sensitive SERS tag of Au@4-MBA@AgCS was successfully prepared.

### 3.4. Characterization of Signal Unit and Capture Unit

**Figure 3:**
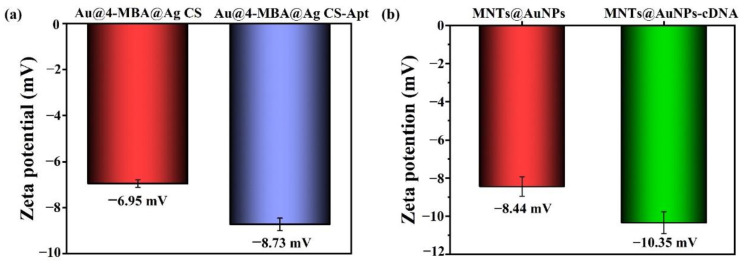
(**a**) Changes in the Zeta potential before and after modification of aptamer by Au@4-MBA@AgCS. (**b**) Changes in the Zeta potential before and after modification of BPA complementary chain by MNTs@AuNPs.

To determine whether the BPA aptamer and its complementary strand were successfully bound to the surfaces of Au@4-MBA@AgCs and MNTs@AuNPs, we evaluated the Zeta potential. After the incubation of the cDNA with MNTs@AuNPs, the average Zeta potential was reduced from −8.44 mV to −10.35 mV because of the phosphate group’s negative charges following hydrolysis (Figure 3b). Furthermore, the average Zeta potential decreased from −6.95 mV to −8.73 mV, as shown in Figure 3a, owing to the conjunction of the aptamer and the Au@4-MBA@AgCS particles through the Au–S coordination. These results indicated that the reporter (Au@4-MBA@AgCS-Apt) and capture units (MNTs@AuNPs-cDNA) were obtained and prepared for SERS detection of BPA based on the proposed strategy.

### 3.5. Optimization of Detection Conditions

To obtain the highest sensitivity and lowest detection limit for BPA sensing, the best detection conditions were explored. In this study, the reaction temperature of the sensors was set to 25 °C. 

**Figure 4:**
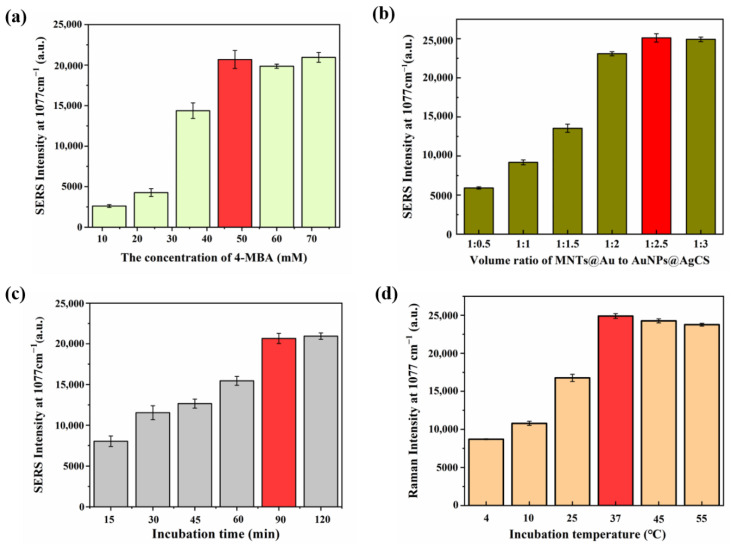
SERS intensity at 1077 cm^−1^ for (**a**) various 4-MBA concentrations (10, 20, 30, 40, 50, 60, 70 mM); (**b**) ratios of different volumes of MNTs@AuNPs to Au@AgCS (1:0.5, 1:1, 1:1.5, 1:2, 1:2.5, 1:3); (**c**) various incubation periods (15, 30, 45, 60, 90, 120 min); and (**d**) different incubation temperatures. The standard deviation of the repeated experiments is indicated by the error bars.

The preparation conditions of Au@4-MBA@AgCS were critical for amplifying the SERS signal in the aptamer sensor. To evaluate the Raman signal enhancement effects of the SERS tags, different concentrations (10, 20, 30, 40, 50, 60, and 70 mM) of 4-MBA were investigated. As indicated in Figure 4a, Au@4-MBA@AgCS achieved the maximum Raman enhancement at 1077 cm^−1^ Raman shift when the concentration of 4-MBA was 50 mM. Therefore, a concentration of 50 mM 4-MBA was used in the experiments. 

The sensors constructed with different volume ratios (1:0.5, 1:1, 1:1.5, 1:2, 1:2.5, and 1:3) of the capture probe to the signal probe are important factors that affect the sensitivity and reproducibility of the SERS sensing system. With the assistance of magnetic separation, the Raman intensities of the supernatants were measured. As shown in Figure 4b, the optimized volume ratio of the capture probe to the signal probe is 1:2.5.

Based on this ratio, the SERS spectra for various incubation periods (15, 30, 45, 60, 90, and 120 min) of the capture and signal probes were measured and analyzed (Figure 4c). The SERS signal intensity gradually increased with an increase in incubation time, and the strongest signal was observed after 90 min of incubation. The intensity of the Raman signal remained unchanged after 90 min of incubation.

Furthermore, we monitored the impact of the Raman signal at five temperatures (4, 25, 37, 45, and 55 °C) to characterize the optimal incubation temperatures for the preparation of the core–satellite assemblies (Figure 4d). The incubation temperature was optimized, and the results showed that the best Raman enhancement performance was achieved at 37 °C. Consequently, a mixing ratio of 1:2.5 capture probe to signal probe at 37 °C for 90 min was chosen as the optimal condition for the construction of the SERS aptasensor.

### 3.6. BPA Detection

**Figure 5:**
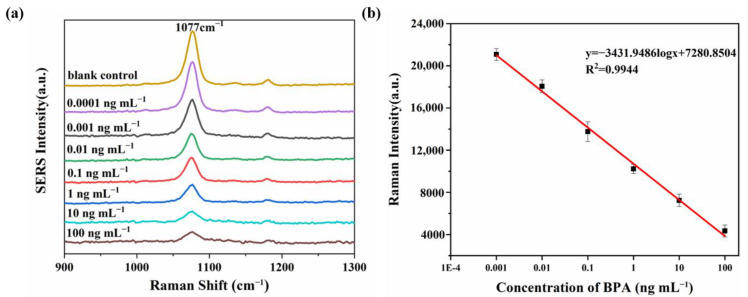
(**a**) Signal intensities of the different concentrations of BPA (from top to bottom 0, 0.0001, 0.001, 0.01, 0.1, 1, 10, 100 ng mL^−1^) in the surface-enhanced Raman spectra. (**b**) Calibration curve. Error bars indicate the standard deviation of the repeated experiments.

The overall SERS intensity was investigated by adjusting the settings for various BPA concentrations using the designed SERS aptamer sensor (Figure 5). 

Figure 5a shows the SERS signal intensities of the nanocomposites after magnetic separation in response to varying BPA concentrations. The SERS responses of the aptasensor in the competitive assay of free BPA were observed, and the SERS signal intensities were dynamically reduced with an increase in concentrations of BPA within the range of 0.001–100 ng mL^−1^. The calculated linear regression equation was y = −3431.9486logx + 7280.8504, having a correlation coefficient R2 = 0.9944 (Figure 5b) and LOD = 0.75 pg mL^−1^ for BPA. The X value is the concentration of BPA, and the Y value is the intensity of the Raman peak at 1077 cm^−1^. The SERS aptamer sensor based on Au@AgCS NPs and MNTs@AuNPs has great potential as a tool for detecting hazardous substances in various samples.

### 3.7. Specificity Evaluation and Real Samples Analysis

The specificity of this assay is owing to its ability to specifically identify and determine a target from various structural analogs, which is an important index for revealing the selectivity and detection accuracy of the biosensor. To evaluate the specificity of the SERS assay strategy for BPA detection under the same experimental conditions, control experiments were carried out using other potential interferents, including E2, 4-tBP, DOD, and DES. As illustrated in Figure S6, the SERS spectra showed no obvious changes in response to these structural analogs, whereas the samples with BPA showed a significant decrease in the Raman signal at 1077 cm^−1^. The SERS biosensing platform has excellent selectivity for BPA detection owing to the high bioaffinity and specificity of the aptamers.

Finally, we examined the reliability and accuracy of the SERS biosensing platform using spiked samples. It is well known that BPA is commonly used in the manufacturing of polycarbonate plastics; however, after repeated high-temperature sterilization, the plastic bottles age, resulting in more BPA being released. A PC bottle was selected as the real sample for the analysis. Furthermore, the samples spiked with various concentrations of BPA (1, 10, 50, and 100 ng mL^−1^) were measured using SERS sensors for quantitative analysis. The obtained SERS response was compared with a standard curve to estimate the BPA concentration in the samples. The results were verified using HPLC and are summarized in Table 1. The results obtained using the SERS aptasensors showed a good correlation with those obtained using HPLC. The recovery of the spiked samples ranged from 96.97% to 113.40%, and the RSD ranged from 2.92% to 6.47%, indicating that our SERS sensors have great potential for the detection of BPA in real samples.

## 4. Conclusions

In summary, a highly sensitive and selective SERS aptasensor was fabricated and used for BPA detection. We innovatively integrated interference-free signal units and magnetic separation into a competing system to form core–satellite nanoassemblies, which contributed to the enhanced SERS signals. This work has shown a broad linear range of 0.001–100 ng mL^−1^, having an R^2^ value of 0.9944 and a detection limit of 0.75 pg mL^−1^. Structural analogs of BPA, such as E2, 4-tBP, DOD, and DES, could have an impact on the aptamer sensor’s specificity and sensitivity. However, these structural analogs have an insignificant effect on the sensing system, indicating high selectivity. Thus, SERS aptamer sensors can be used to evaluate food safety when checking for BPA contamination. The potential of SERS biosensors can be expanded by varying the aptamer probe and signal probe, and the potential of the SERS biosensor can be expanded for the detection of other hazardous substances. The prepared SERS aptasensor exhibited remarkable Raman signal enhancement and excellent SERS sensitivity. Therefore, the proposed aptasensor offers a broad range of applications in the food safety and environmental health sectors.

## Figures and Tables

**Table 1:** Detection of BPA in spiked samples (*n* = 5).

Samples	Spiked Concentration (ng mL^−1^)	HPLC			SERS Sensors		
		Detection Value (ng mL^−1^) Mean ± SD	Recovery Rate (%)	RSD (%)	Detection Value (ng mL^−1^) Mean ± SD	Recovery Rate (%)	RSD (%)
PC bottle	0	45.3 ± 0.6	-	0.9	44.7 ± 1.7	-	3.2
	1	45.7 ± 0.4	87.0	0.3	45.9 ± 2.4	104.3	4.1
	10	55.2 ± 0.7	104.2	1.1	46.3 ± 2.9	113	4.4
	50	96.1 ± 0.9	100.7	0.8	93.5 ± 4.3	96.9	6.4
	100	145.6 ± 0.9	100.5	0.4	144.8 ± 1.8	99.8	2.9

“–”: not detected.

## Data Availability

Not applicable.

## Supplementary Materials

The following supporting information can be downloaded at https://www.mdpi.com/article/10.3390/bios12060387/s1, Figure S1: Hysteresis curves for MNTs@AuNPs; Figure S2: FT-IR spectra of HNTs, MNTs, and MNTs-NH_2_; Figure S3: Zeta potential of HNTs, MNTs, and MNTs-NH_2_ and MNTs@AuNPs; Figure S4: X-ray diffraction patterns of MNTs and MNTs@AuNPs; Figure S5: (a) X-ray photoelectron spectra of MNTs and MNTs@AuNPs. (b–g) XPS scan curves for the Au, C, N, O, Si, and Al in MNTs@AuNPs; Figure S6: Comparison of the Raman peak intensities at 1077 cm^−1^ for estradiol (E2), 4-tert-butylpyridine (4-tBP), 4,4′-dihydroxybiphenyl (DOD), diethylstilbestrol (DES), and bisphenol A (BPA) using the reporter probes.
