# Supplementary material for: Magnetic Halloysite Nanotube-Based SERS Biosensor Enhanced with Au@Ag Core–Shell Nanotags for Bisphenol A Determination
Source: Biosensors (Basel). 2022 Jun 2;12(6):387. doi: 10.3390/bios12060387 (PMC9221462; doi:10.3390/bios12060387)
Supplement: Supplementary file 1 [file biosensors-12-00387-s001.zip › biosensors-1733844-supplementary.pdf]

Supplementary Files

Magnetic Halloysite Nanotubes based SERS Biosensor Enhanced with Au@Ag Core-Shell Nanotags for Bisphenol A Determination

Sen Li,[a], Defu He[a,b], Shuning Li[a,c], Ruipeng Chen[a,c], Yuan Peng[a], Shuang Li[a], Dianpeng Han [a], Yu Wang[a]， Kang Qin[a]， Shuyue Ren[a]， Ping Chen[b*]， Zhixian Gao[a*]

[a] Tianjin Key Laboratory of Risk Assessment and Control Technology for Environment and Food Safety, Tianjin Institute of Environmental and Operational Medicine, Tianjin 300050, China

[b] School of Food Science and Engineering, Jilin Agricultural University, Changchun 130118, China

[c] State Key Laboratory of Food Nutrition and Safety, Tianjin University of Science & Technology, Tianjin 300457, China

* Corresponding Author: gaozhx@163.com; ccchenping@sina.com

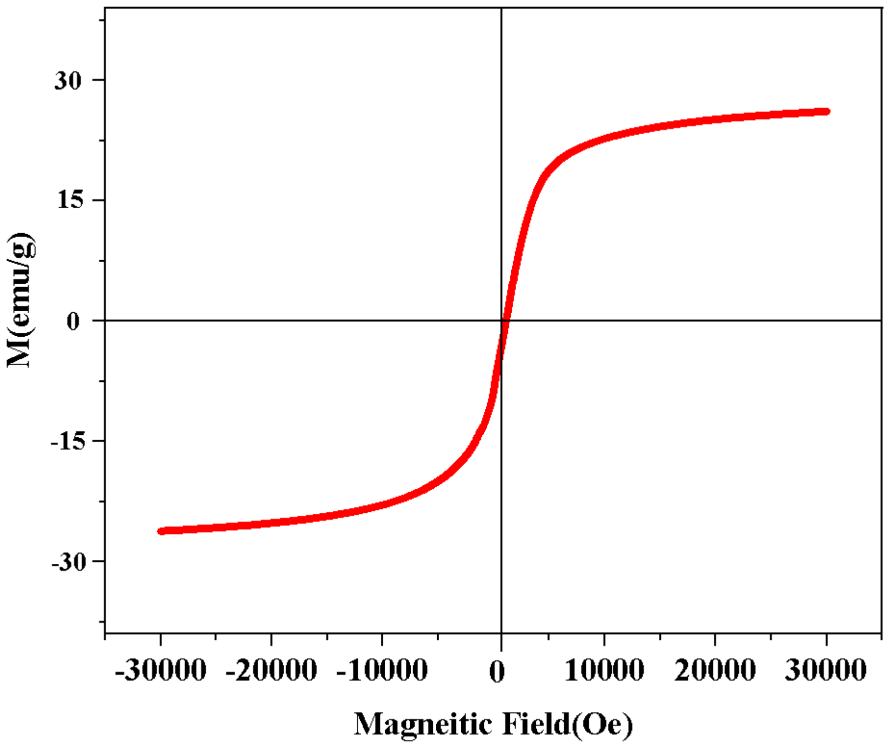

Figure S1. Hysteresis curves for MNTs@AuNPs.

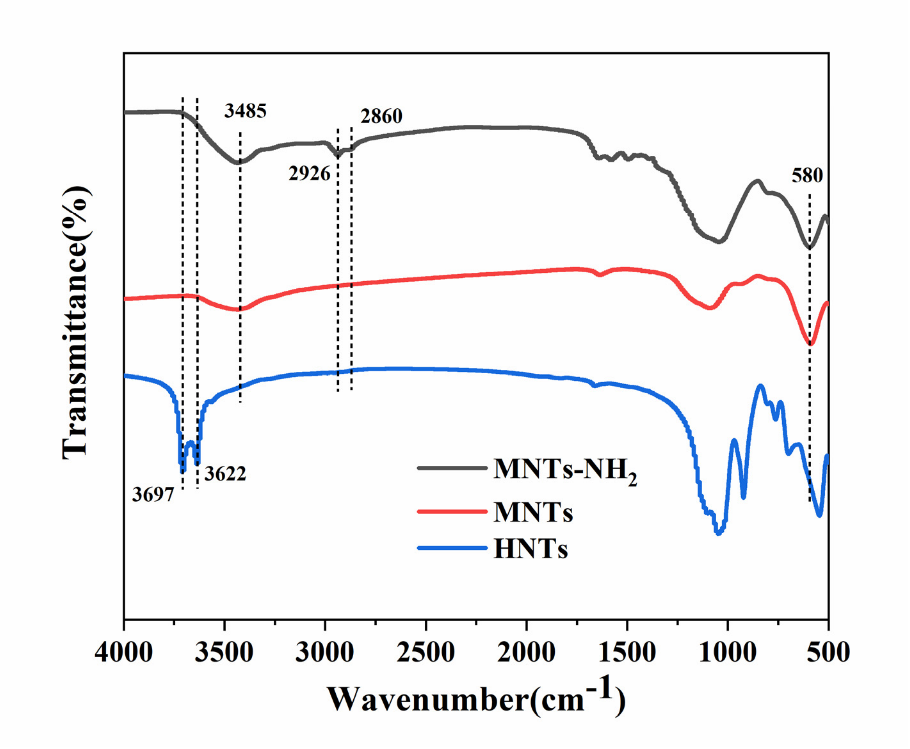

Figure S2. FTIR spectra of HNTs, MNTs and MNTs-NH_2.

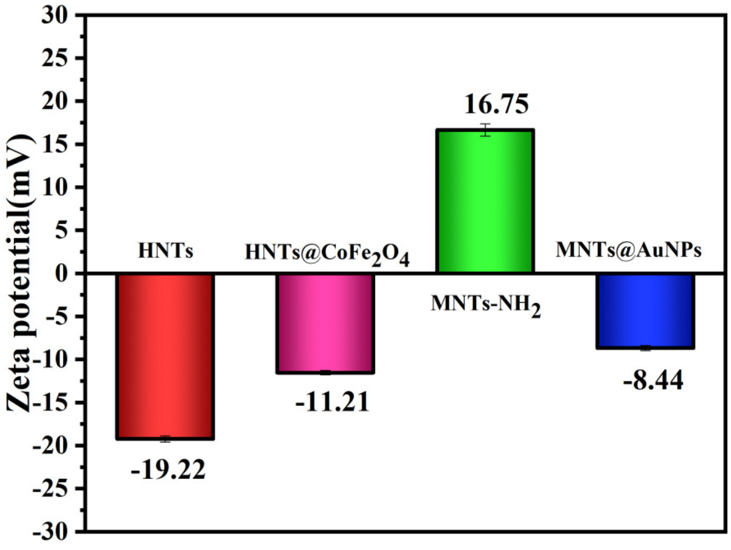

Figure S3. Zeta potential of HNTs, MNTs and MNTs-NH$_2$ and MNTs@AuNPs.

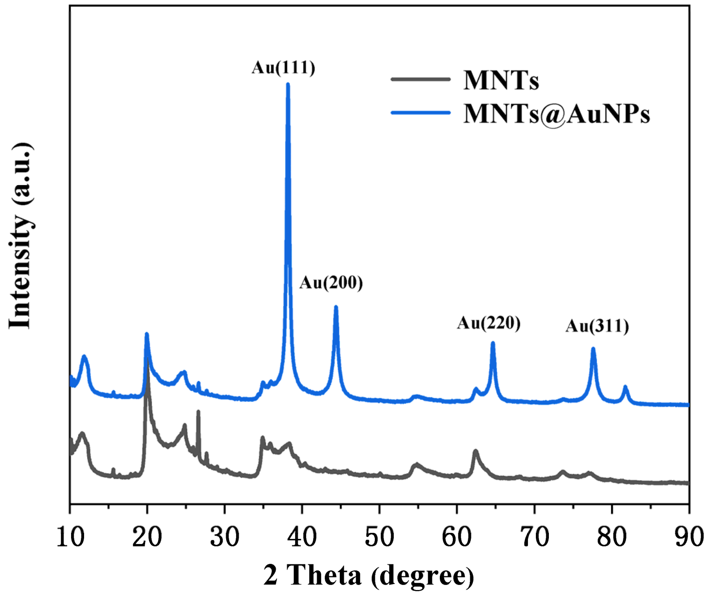

Figure S4. X-ray diffraction patterns of MNTs and MNTs@AuNPs.

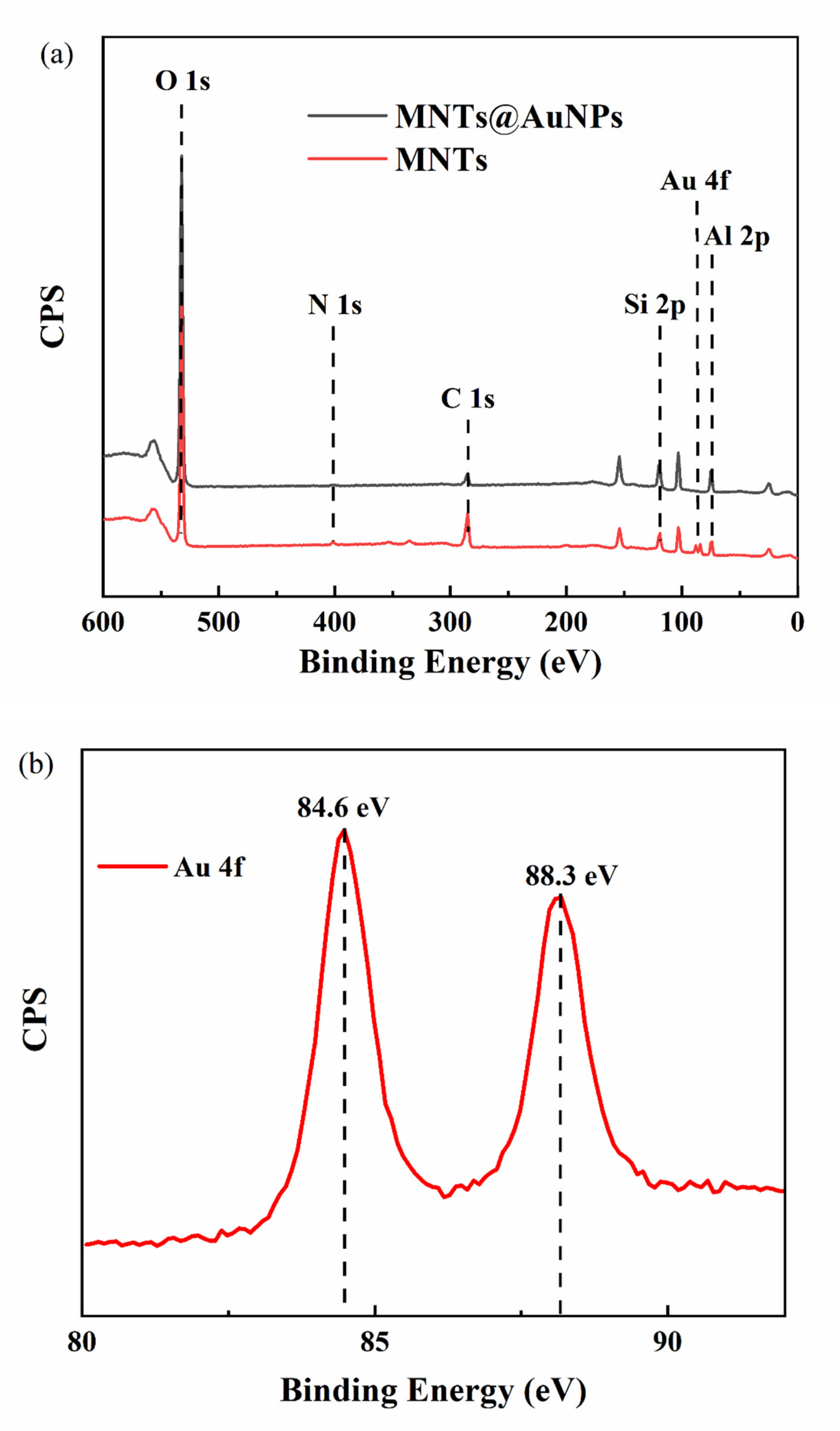
(a)
O 1s
MNTs@AuNPs
MNTs
Au 4f
Al 2p
N 1s
Si 2p
C 1s
CPS
600
500
400
300
200
100
0
Binding Energy (eV)
(b)
84.6 eV
88.3 eV
Au 4f
CPS
80
85
90
Binding Energy (eV)

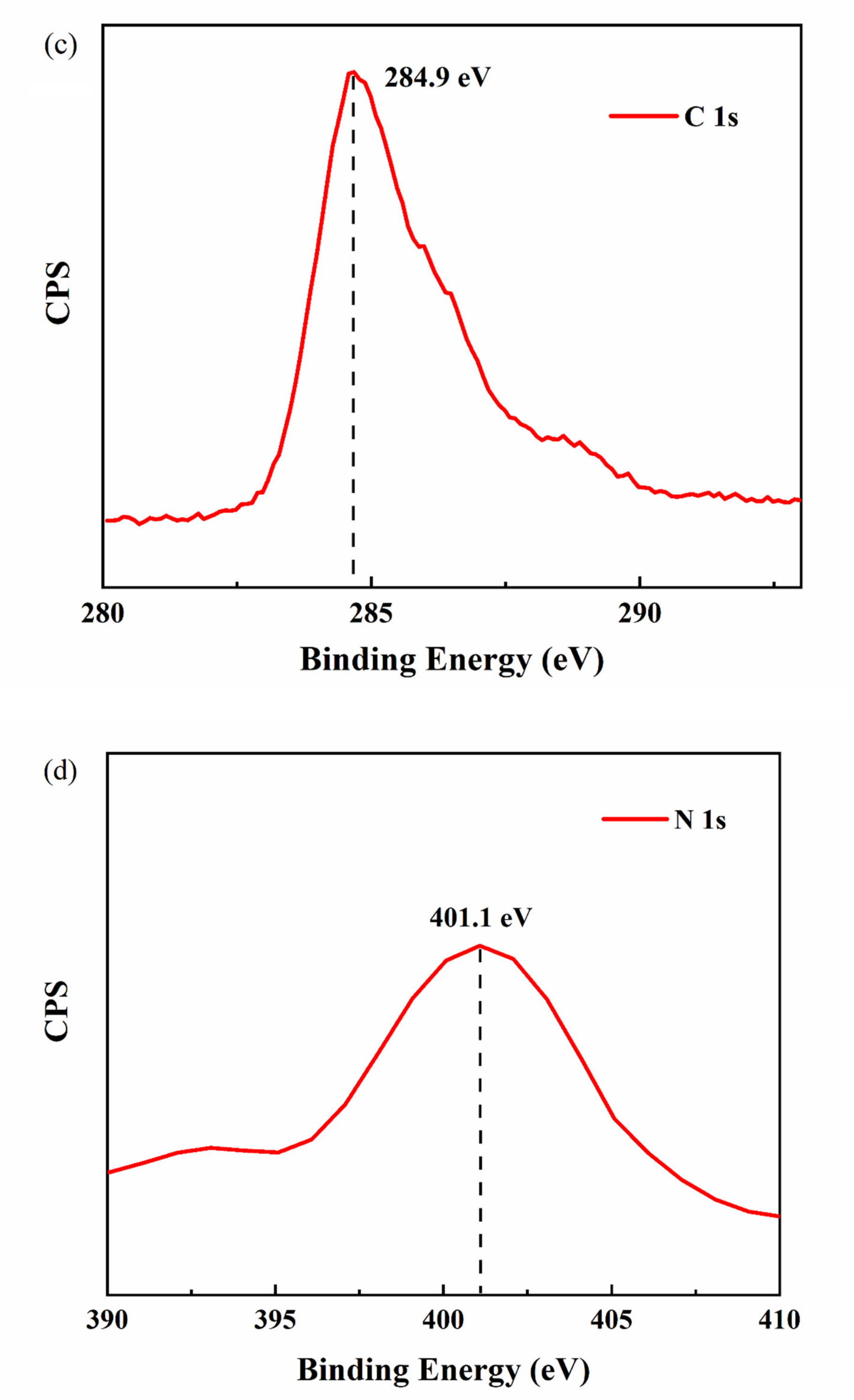
(c)
284.9 eV
C 1s
CPS
280
285
290
Binding Energy (eV)
(d)
N 1s
401.1 eV
CPS
390
395
400
405
410
Binding Energy (eV)

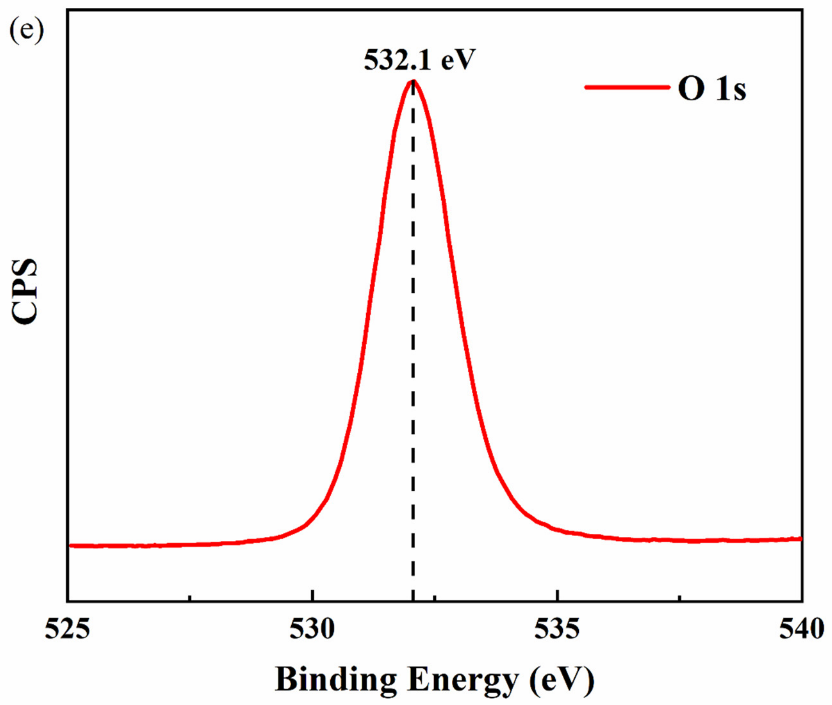
(e)
532.1 eV
O 1s
CPS
525
530
535
540
Binding Energy (eV)

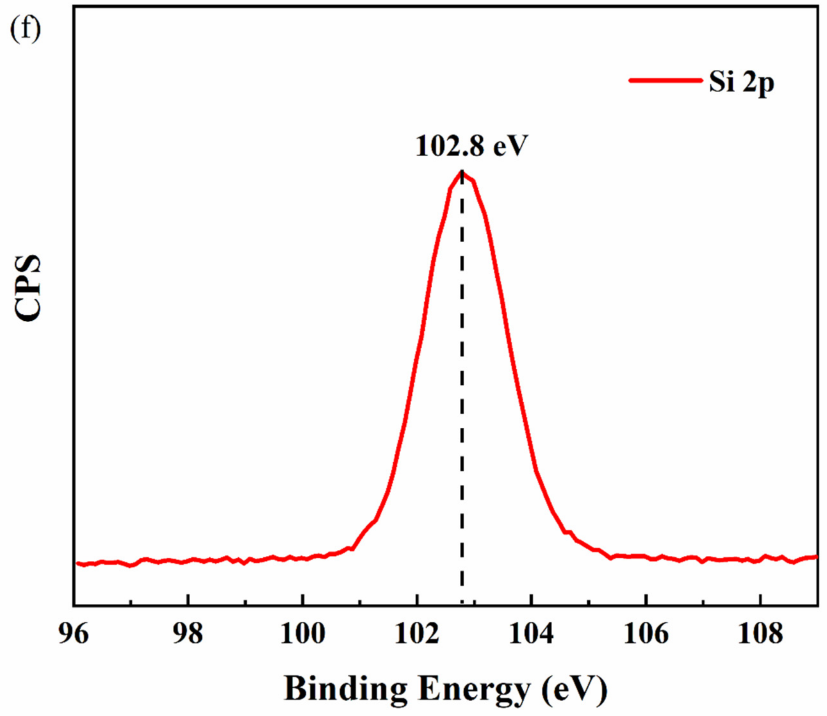
(f)
Si 2p
102.8 eV
CPS
96
98
100
102
104
106
108
Binding Energy (eV)

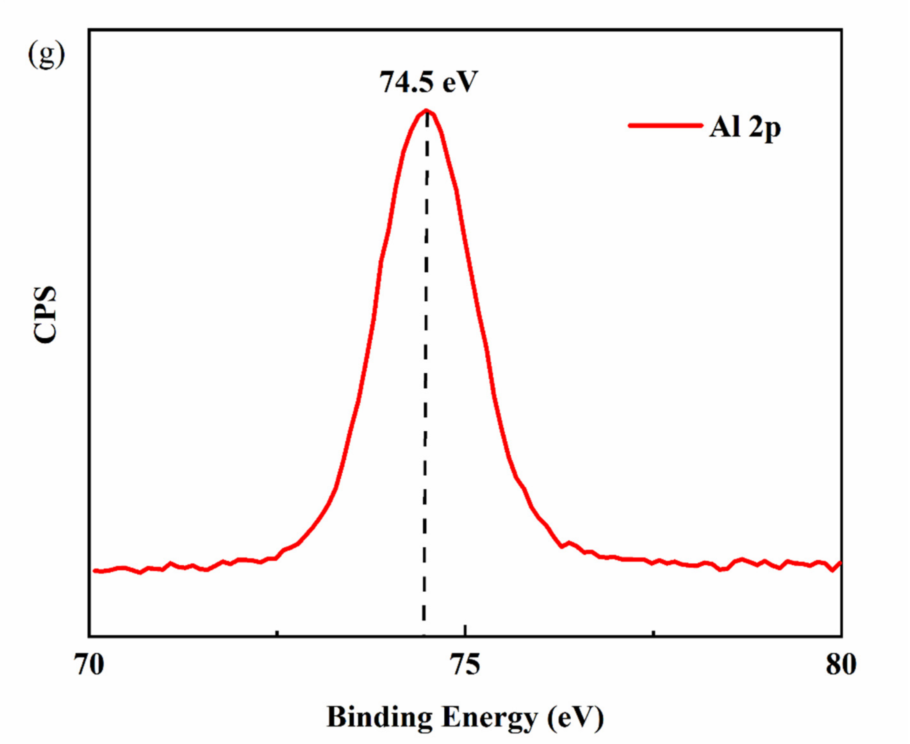

Figure S5. (a) X-ray photoelectron spectra of MNTs and MNTs@AuNPs. (b-g) XPS scan curves for the Au, C, N, O, Si, and Al elements in MNTs@AuNPs.

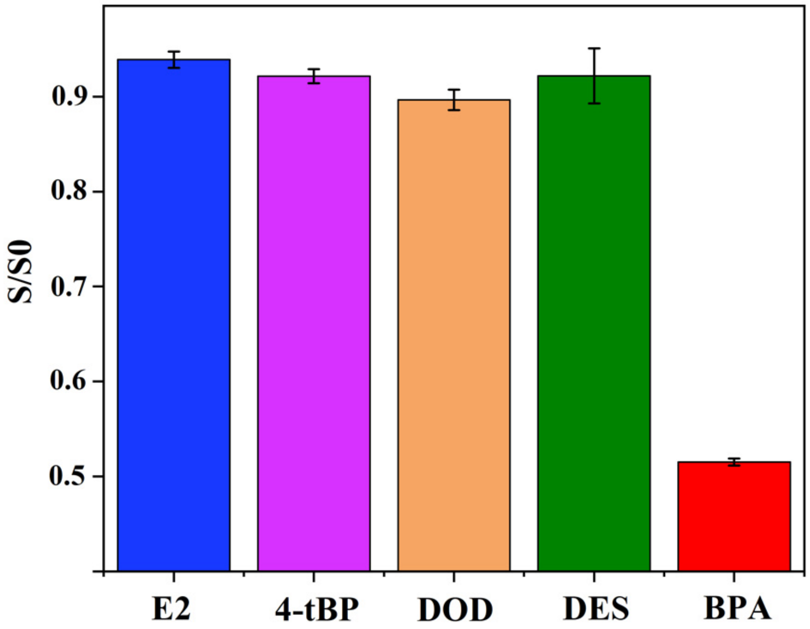

Figure S6. Comparison of Raman peak intensities at 1077 cm^{-1} for estradiol (E2), 4-tert-butylpyridine (4-tBP), 4,4'-dihydroxybiphenyl (DOD), hexenestrol (DES), and bisphenol A (BPA) by the reporter probes. The SERS intensities of the experimental group (S) and the control group (S0) were detected. Error bars indicate the standard deviation of the three replicate experiments.
